# Mutations in human *C2CD3* cause skeletal dysplasia and provide new insights into phenotypic and cellular consequences of altered C2CD3 function

**DOI:** 10.1038/srep24083

**Published:** 2016-04-20

**Authors:** Claudio R. Cortés, Aideen M. McInerney-Leo, Ida Vogel, Maria C. Rondón Galeano, Paul J. Leo, Jessica E. Harris, Lisa K. Anderson, Patricia A. Keith, Matthew A. Brown, Mette Ramsing, Emma L. Duncan, Andreas Zankl, Carol Wicking

**Affiliations:** 1 Institute for Molecular Bioscience, The University of Queensland, Brisbane, QLD 4072, Australia; 2 The University of Queensland Diamantina Institute, University of Queensland, Australia; 3 Department of Clinical Genetics, Aarhus University Hospital, Denmark; 4 Queensland University of Technology (QUT), Institute of Health and Biomedical Innovation (IHBI), Queensland, Australia; 5 Department of Pathology, Aarhus University Hospital, Denmark; 6 Department of Endocrinology, James Mayne Building, Royal Brisbane and Women’s Hospital, Butterfield Road, Herston, QLD 4029, Australia; 7 Academic Department of Medical Genetics, Sydney Children’s Hospitals Network (Westmead), Westmead, NSW 2145, Australia; 8 Childrens Hospital Westmead Clinical School, Sydney Medical School, The University of Sydney, NSW 2145, Australia; 9 Garvan Institute of Medical Research, Darlinghurst NSW 2010, Australia

## Abstract

Ciliopathies are a group of genetic disorders caused by defective assembly or dysfunction of the primary cilium, a microtubule-based cellular organelle that plays a key role in developmental signalling. Ciliopathies are clinically grouped in a large number of overlapping disorders, including the orofaciodigital syndromes (OFDS), the short rib polydactyly syndromes and Jeune asphyxiating thoracic dystrophy. Recently, mutations in the gene encoding the centriolar protein C2CD3 have been described in two families with a new sub-type of OFDS (OFD14), with microcephaly and cerebral malformations. Here we describe a third family with novel compound heterozygous *C2CD3* mutations in two fetuses with a different clinical presentation, dominated by skeletal dysplasia with no microcephaly. Analysis of fibroblast cultures derived from one of these fetuses revealed a reduced ability to form cilia, consistent with previous studies in *C2cd3*-mutant mouse and chicken cells. More detailed analyses support a role for C2CD3 in basal body maturation; but in contrast to previous mouse studies the normal recruitment of the distal appendage protein CEP164 suggests that this protein is not sufficient for efficient basal body maturation and subsequent axonemal extension in a C2CD3-defective background.

The primary cilium is a single microtubule (MT)-based organelle that extends from the surface of most quiescent vertebrate cells. The cilium has both mechano- and chemosensory functions, and has been linked to the regulation of a number of signalling pathways, including the hedgehog (HH), WNT, planar cell polarity (PCP) and (possibly) calcium pathways^1^,^2^,^3^,^4^,^5^.

The assembly of the primary cilium is dependent on the docking of the mother centriole to the cell membrane and its conversion to a basal body, which anchors the cilium in the cell. In some cell types this involves the binding of a ciliary vesicle to the distal end of the mother centriole, with subsequent docking to the cell membrane mediated by distal appendages present specifically on the mother centriole^6^. Before extension of the ciliary axoneme can occur, the distal end of the mother centriole is uncapped by removal of the protein CP110^7^, with recent data suggesting that this occurs prior to ciliary vesicle binding^8^. Following plasma membrane docking, the axoneme is extended from the basal body through the action of a specialised bi-directional motor-driven trafficking system known as intraflagellar transport or IFT^9^.

Defects in the assembly or function of the primary cilium are associated with a heterogeneous group of disorders, termed ciliopathies. Ciliopathies are characterised by an overlapping series of clinical features, including kidney and liver cysts, retinal degeneration, craniofacial dysmorphologies, polydactyly, brain defects and chondrodysplasia^10^. Orofaciodigital syndromes (OFDS, MIM 311200) are a sub group of ciliopathies that primarily affect the oral cavity, face and digits, with additional features variably present in the skeleton, kidneys, heart and brain^11^. Based largely on the presence of these additional features, multiple clinical subtypes of OFDS have been described, with considerable phenotypic overlap between subtypes. The skeletal ciliopathies are a subgroup of ciliopathies that are primarily characterized by their skeletal features. This group includes the short-rib polydactyly syndromes (SRPS; MIM 613091, 263520), Jeune asphyxiating thoracic dystrophy (JATD; MIM208500) and Ellis van Creveld syndrome (EVC; MIM 225500). There is considerable overlap between the different OFDS and skeletal ciliopathy subgroups. For example, OFD4 (Mohr-Majewski syndrome; MIM258860) shares characteristics of both OFD2 (Mohr syndrome, MIM 252100) and SRPS II (Majewski syndrome, MIM263520)^12^,^13^.

To date mutations in several genes have been identified in OFDS individuals, including those that encode the centriolar protein OFD1 in X-linked OFD1^14^, the transition zone proteins TMEM231 in OFD3^15^ and Tectonic3 (TCTN3) in OFD4^16^, the DEAD-box-containing RNA helicase protein DDX59 in OFD5^17^, and TMEM216 and C5ORF42 in OFD6^18^,^19^. Recently, mutations in *C2CD3*, encoding a C2 calcium-dependent domain containing protein, were reported in two families with OFDS^20^. The affected individuals in these families displayed typical features of OFDS, but had additional features including microcephaly and cerebellar anomalies with molar tooth sign. Based on the unique presentation in these two families, a new subtype of OFDS (OFD14; MIM615948) was suggested^20^.

*C2cd3* was first identified in a forward genetic screen for mutations affecting mouse embryogenesis, with a presumed hypomorphic splicing mutation in *C2cd3* responsible for the recessive embryonic lethal *Hearty* (*Hty*) allele resulting in exencephaly, loss of laterality, twisted body axis and polydactyly in all limbs^21^. Further analysis showed that *C2cd3*^*Hty/Hty*^ embryos display disrupted HH signalling, and fewer cilia form both *in vivo* in the embryonic node, and *in vitro* in isolated mouse embryonic fibroblasts (MEFs)^21^. More detailed analysis of both *C2cd3*^*Hty/Hty*^ and an additional loss-of-function *C2cd3*-mutant mouse allele (*C2cd3*^*Gt/Gt*^) showed that C2CD3 also acts as a positive regulator of centriolar length, directly opposing the role of its binding partner, the related OFDS protein OFD1^20^. Furthermore, C2CD3 localises to the distal end of centrioles and to surrounding electron-dense granules known as centriolar satellites, and mediates distal appendage assembly, ciliary vesicle docking and recruitment of other ciliary proteins to the mother centriole^22^,^23^. More recently, the chicken mutant *talpid*^2^, which is characterised by a series of dysmorphologies consistent with OFD14, was shown to harbor recessive mutations in *C2cd3*^24^,^25^. Like *C2cd3*-mutant mouse cells, *talpid*^2^ chicken cells show a reduced capacity to ciliate both *in vivo* and in culture, an abnormality thought to arise from defective docking of the mother centriole to the cell membrane^24^.

Using whole exome sequencing, we detected compound heterozygous *C2CD3* mutations in two fetuses from the same family with a different phenotype to that previously reported^20^. We present analysis of fibroblasts from one of these fetuses, providing the first description of the cellular phenotype of human C2CD3-deficient cells. Our data confirm studies in mutant mouse cells, showing inefficient ciliogenesis due to defects in mother centriole uncapping and localisation of the core IFT protein IFT88 to the centrosome. Unlike the mouse cells, however, the mother centriole in human C2CD3-deficient fibroblasts displays correct localisation of the centriolar distal appendage protein CEP164. This suggests that recruitment of CEP164 is necessary but not sufficient for the formation of fully functional distal appendages, a step required for uncapping of the mother centriole, basal body formation and subsequent axonemal extension.

## Results

### *C2CD3* is mutated in a family with a skeletal dysplasia

**Figure 1:**
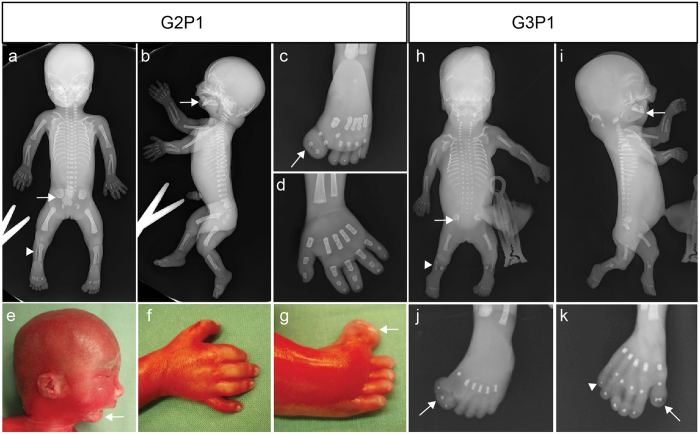
Clinical features of the two subjects in this study. (**a–g**) G2P1; (**h–k**) G3P1. Common features include short ribs, shortened long bones, micrognathia (arrow, **b,e,i**) and trident acetubula (arrow, **a,h**). G2P1 displayed preaxial polydactyly (duplicated hallux) of the feet (arrow, **c,g**), and no polydactyly of the hands, although digits appear short (**d,f**). G3P1 displayed preaxial polydactyly (triplicated hallux) of the feet (arrow **j**), and pre- (arrow, **k**) and postaxial (arrowhead, **k**) polydactyly of the hands. The tibiae in G3P1 were hypoplastic (arrowhead, **h**) but appeared normal in G2P1.

We identified a non-consanguineous family from Lebanon of Palestinian descent, with two consecutive pregnancies complicated by skeletal dysplasia. The first and fourth pregnancies resulted in healthy children. Prenatal ultrasound examination at 20 + 3 weeks of the first affected fetus (G2P1) showed normal biparietal diameter but short tubular bones, polydactyly, renal cysts and Dandy-Walker malformation. The pregnancy was terminated, and subsequent autopsy revealed a female fetus with facial dysmorphism (microphthalmia, hypertelorism, flat and broad nasal bridge, dysplastic and posteriorly rotated ears, micrognathia, and a high arched palate), preaxial polydactyly of the feet presenting as a duplicated and bent hallux, brachydactyly, short ribs, short bent tubular bones, trident acetabula (Fig. 1a–g) and a number of ductal kidney cysts. The anus was anteriorly located, and atypical lung lobation, symmetrical liver, and mobile caecum were observed. The brain was not examined at post-mortem in accordance with parental wishes. In a subsequent pregnancy, an early ultrasound scan at 15 + 2 weeks revealed a male fetus (G3P1) with short and bent tubular bones. Autopsy following termination of pregnancy revealed facial dysmorphism, preaxial polydactyly of the hands and feet (presenting as duplication of the thumb and triplication of the hallux), postaxial polydactyly of the hands, hypoplastic tibia, short ribs, short bent tubular bones, trident acetabula (Fig. 1h–k) and kidney cysts. Unlike G2P1, a minor heart malformation (atrial septal defect) was also detected in G3P1. Lungs were without lobation, liver was symmetrical and caecum was mobile. Micropenis/ambiguous genitalia were also noted. Permission was given for cerebrum autopsy, revealing holoprosencephaly, Dandy-Walker malformation with a dilated cisterna magnum and vermis aplasia, cerebellar hypoplasia, polymicrogyria, and hydrocephalus (not shown). A clinical diagnosis of a skeletal ciliopathy was made, but due to the clinical overlap between various forms of OFDS, SRPS and JATD, a more specific diagnosis could not be established.

**Figure 2:**
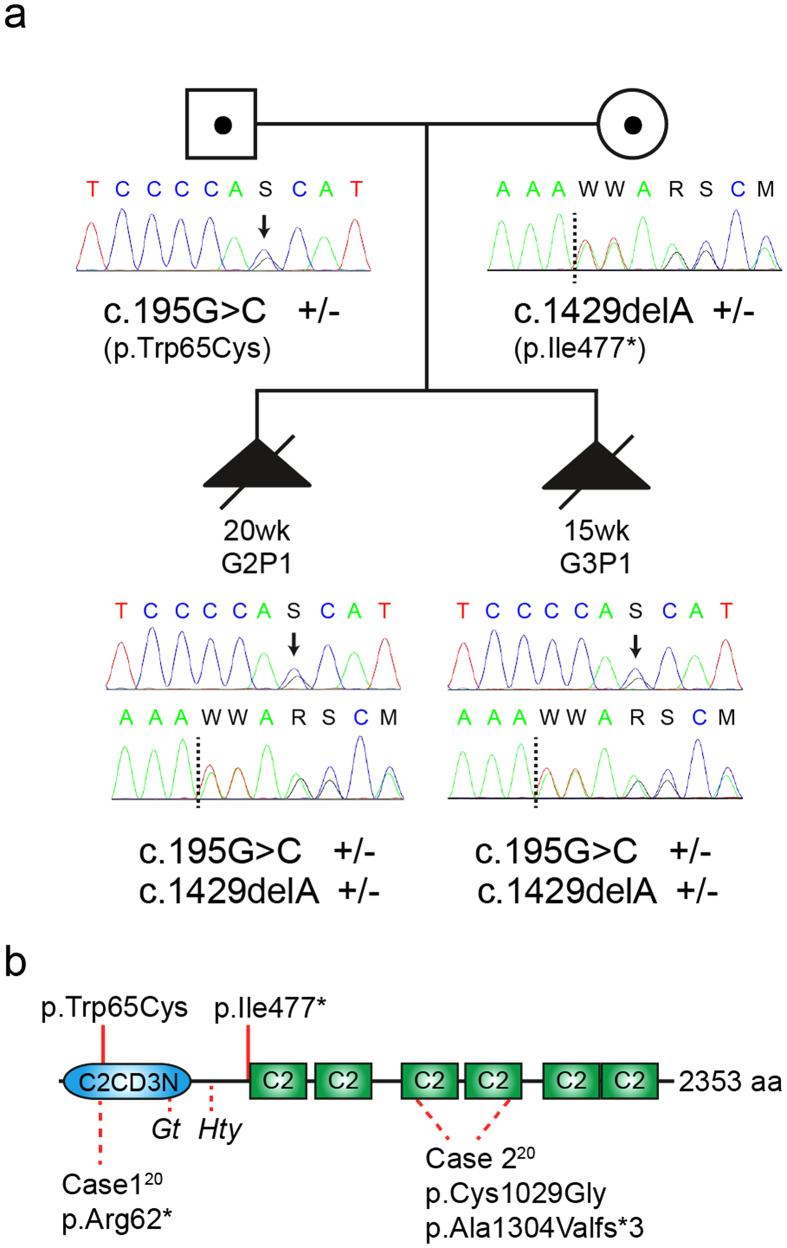
Segregation of *C2CD3* mutations, and protein ideogram indicating location of variants. (**a**) *C2CD3* variants present in the parents and affected individuals in the reported family. Sequence reads highlight genotype for variants identified by Sanger sequencing under each individual. Arrows denote missense mutation, dotted lines denote deletion site. Note that the sequence scan for the c.195G > C mutation shows the reverse strand sequence; the c.1429delA mutation is shown on the forward strand. (**b**) Human C2CD3 protein schematic showing mutations detected in the present study (red lines, top), mutations reported in previously reported OFDS cases^20^ (dotted lines, bottom) and mouse *C2cd3* alleles *C2Cd3*^*Gt*^ (*Gt)* and *Hearty* (*Hty*) (dotted lines, bottom). Note the *C2cd3*^*Gt*^ mutation is predicted to result in a truncated protein encoding the N-terminal 161 amino acids only; aberrant splicing in *Hty* leads to proteins either truncated at amino acid 235, or with small in-frame deletions in the same region^20^,^21^. C2: canonical C2 domains, C2CD3N: non-canonical globular C2 domain^29^.

DNA from individual G3P1 was subject to whole exome sequencing. After filtering for quality control, seven genes (*FAM160B1, FCF1, OVGP1, TTN, KIA1109, PDK4,* and *C2CD3*) contained two or more variants that were either novel or rare (minor allele frequency <0.005) and of potential functional consequence, thus fitting compound heterozygous inheritance (See Table 1). Using the same parameters, two genes contained homozygous variants (*FRMPD2* and *PTGFRN*), but this pattern of inheritance was considered less likely in a non-consanguineous family with a rare disease. Of the genes identified, only *C2CD3* was known to be involved in ciliogenesis, with mutations known to cause a ciliopathy phenotype in both humans and animal models^20^,^21^,^23^,^24^. We did not detect any single heterozygous good quality rare (MAF < 0.005) coding/splice site variant in any other known ciliopathy or OFD gene; thus it is unlikely compound heterozygous mutations in another ciliopathy gene were missed through poor coverage. Sanger sequencing confirmed appropriate segregation of the *C2CD3* variants in the parents and affected individuals in this family (Fig. 2a).

Neither of the *C2CD3* variants detected has been previously reported. The first is a missense mutation in exon 2 (c.195G > C: p.Trp65Cys; NM_015531) in a highly conserved nucleotide (genomic evolutionary rate profiling score 5.57), predicted to be deleterious by Polyphen^26^ (score 1.00), SIFT^27^ (score 0.00) and Mutation Taster^28^ (score 0.999). The second variant introduces a STOP codon in a highly conserved region of exon 9 (c.1429delA: p.Ile477*, NM_015531). *C2CD3* encodes a protein with multiple C2 domains, 5–6 at the carboxyl terminus and one novel, non-canonical C2 domain at the amino terminus^20^,^29^ (Fig. 2b). C2 domains have been shown to be involved in protein-protein interactions of other ciliopathy proteins that localise to the transition zone, which lies distal to the basal body^29^,^30^. The STOP mutation (p.Ile477*) is predicted to result in a truncated protein lacking all C-terminal C2 domains, and the missense mutation (p.Trp65Cys) falls within the N-terminal non-canonical C2 domain (Fig. 2b).

### *C2CD3*-mutant cells show defects in the early stages of ciliogenesis

**Figure 3:**
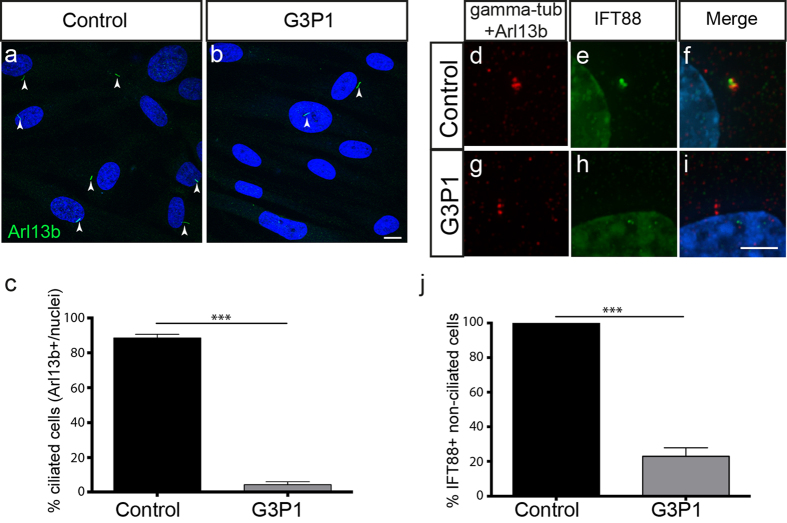
Ciliogenesis and IFT protein recruitment is impaired in fibroblasts isolated from G3P1. (**a,b**) Images showing a lower percentage of cilia (as marked by Arl13b staining) relative to DAPI-stained nuclei in serum-starved *C2CD3*-mutant fibroblast cultures (G3P1), compared to control juvenile dermal fibroblasts. Scale bar = 10 μm. Quantification is shown in (**c**). (**d–i**) Representative images of IFT88 staining (green) in non-ciliated cells (indicated by lack of red Arl13b staining along the axoneme) in serum-starved cultures. IFT88 localises to the centrosome (stained with gamma-tubulin in red) in control fibroblasts (**d–f**) whereas in fibroblasts isolated from G3P1, IFT88 is absent from most centrosomes (**g–i**). Scale bar = 5 μm. (**j**) Quantification of percentage of non-ciliated cells with IFT88-postive centrosomes in cultured *C2CD3*-mutant fibroblasts, relative to normal juvenile fibroblasts. ***p < 0.0001, error bars show SEM. All ciliated cells show normal localisation of IFT88 in both control and mutant cultures, reflecting the requirement for IFT88 in ciliogenesis.

While the effect of *C2CD3* mutations has previously been investigated in mouse and chicken cells^20^,^21^,^24^, direct analysis of *C2CD3*-mutant human cells has not been reported. We had access to human fibroblasts from individual G3P1, and investigated their ability to ciliate following serum starvation to induce ciliogenesis. We found a significant reduction in the percentage of G3P1 cells with a cilium when compared to control juvenile dermal fibroblasts, following staining of the axoneme with either an Arf-like small GTPase ARL13b (Fig. 3a–c) or acetylated α-tubulin antibody (not shown). These data are consistent with previous studies in mouse^21^ and chicken cells^24^. Note that we did not have access to a normal fetal fibroblast control but did confirm that the level of ciliogenesis in a fibroblast line derived from a 14-week gestation fetus with an unrelated non-ciliopathy disease (fetal akinesia), is equivalent to that in the juvenile fibroblast line used as a control in these studies (see Supplementary Fig. S1). This suggests that ciliogenesis in fibroblasts is not affected by the age at which they are derived.

**Figure 4:**
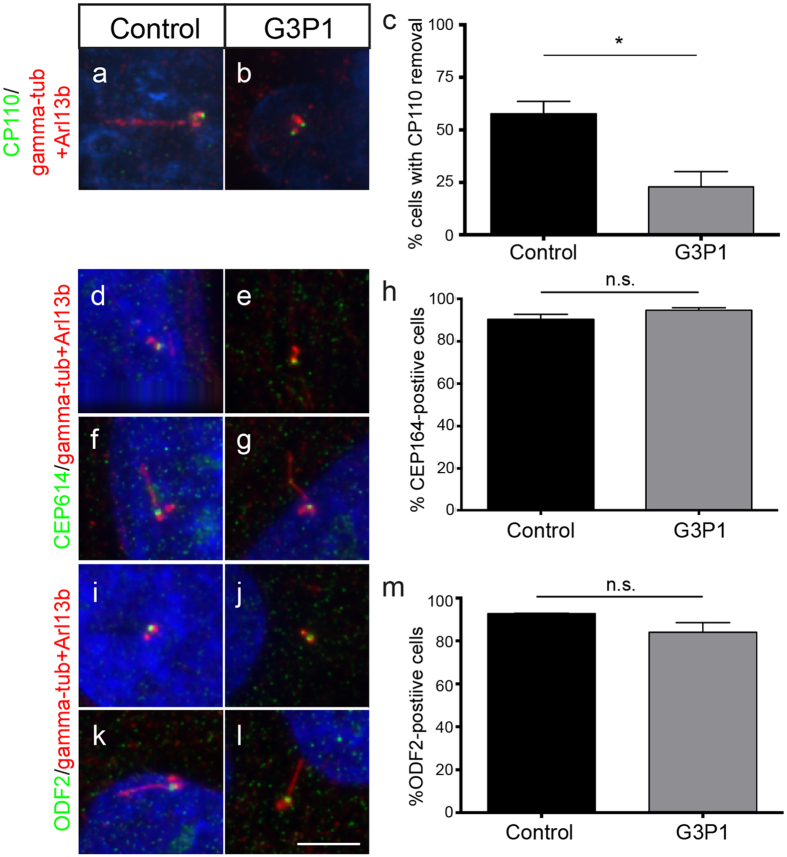
Centriolar appendage and basal body maturation in *C2CD3*-mutant fibroblasts. (**a,b**) Images of CP110 staining (green) in serum starved control and G3P1 fibroblasts. Note that the image in panel (**b**) with CP110 at both centrioles is representative of approximately 77% of cells in G3P1cultures (23% of cells have CP110 removed from the mother centriole, i.e. show staining at just one centriole). This quantification is shown graphically in (**c**) where random cells across both mutant and control cultures with 2 distinct centriolar spots were scored for removal of CP110 from the mother centriole. CP110 is removed in fewer mutant cells relative to control cells. *p < 0.05. (**d–g**) Representative images of staining for the distal appendage marker CEP164 (green) in non-ciliated (**d,e**) and ciliated cells (**f,g**). (**h**) Quantification of cells with CEP164-positive mother centriole or basal body. (**i–l**) Representative images of staining for the sub-distal appendage marker ODF2 (green) in non-ciliated (**i–j**) and ciliated cells (**k,l**). (**m**) Quantification of cells with ODF2-positive mother centriole or basal body. Scale bar = 5 μm. In all cases the centrosome is marked with gamma-tubulin and the axoneme with Arl13b, both in red. n.s. not significant, error bars show SEM.

Defective axonemal extension is a common consequence of altered IFT, and accordingly *C2cd3*-mutant mouse cells fail to recruit a number of IFT proteins to the centrosome^23^. The core anterograde IFT protein IFT88 is essential for ciliogenesis and is normally localised to centrioles prior to cilia assembly, and to the transition zone and along the axoneme in ciliated cells^31^. Consistent with the studies in mouse cells, we observed a defect in the localisation of IFT88 at the centrioles of non-ciliated serum-starved G3P1 mutant cells (Fig. 3d–j), while all ciliated cells recruited IFT88 normally. In addition to IFT, axonemal extension is also dependent on uncapping of the distal end of the mother centriole by removal of CP110^7^. CP110 removal is defective in *C2cd3*-mutant mouse cells^23^, and in agreement with this we found that CP110 was not efficiently removed from the mother centriole in serum-starved G3P1 cells relative to control fibroblast cultures (23% cells in mutant cultures versus 58% in control cultures show CP110 removal; i.e CP110 at only one centriole; Fig. 4a–c). This suggests that *C2CD3*-mutant G3P1 cells do not efficiently uncap the mother centriole, one of the earliest steps in ciliogenesis, even when ciliogenesis is induced by serum starvation.

CP110 removal is thought to depend on functional distal appendages, and was assumed to occur after plasma membrane docking of the mother centriole^32^, although recent data suggests that CP110 removal may actually occur prior to membrane docking^8^. Previously reported analysis of MEFs derived from both *C2cd3*^*Hty/Hty*^ and *C2cd3*^*Gt/Gt*^ embryos revealed a dramatic reduction in the localisation of the distal appendage protein CEP164 to the mother centriole^20^. We were therefore surprised to find that CEP164 was normally localised to the mother centriole and/or basal body in most ciliated and non-ciliated G3P1 cells (Fig. 4d–h), suggesting normal localisation of at least some distal appendage proteins. Another key feature of mother centrioles are the subdistal appendages, which serve a distinct function to distal appendages by stabilising microtubules^33^. We stained for the subdistal appendage protein ODF2 and found that ODF2 was also localised to the mother centriole and/or basal body in G3P1 cells at a similar frequency to control cells (Fig. 4i–m). Like the CEP164 data, this is in contrast to mouse *C2cd3*-mutant cells, where both *C2cd3*^*Hty/Hty*^ and *C2cd3*^*Gt/Gt*^ MEFs show defective localisation of ODF2, suggesting the absence of subdistal appendages, supported by transmission electron microscopy (TEM) analysis^20^. However, an alternative analysis in *C2cd3*^*Gt/Gt*^ MEFs suggested the presence of normal subdistal appendages both by TEM and staining for the markers ninein and pericentrin^23^. This suggests either that C2CD3 may regulate the recruitment of only a subset of subdistal appendage proteins, or the differences may result from different experimental conditions in the two published studies.

Our data suggest that, like *C2cd3*-mutant mouse cells, the human cells are defective in their ability to uncap the mother centriole and correctly localise IFT88, thus blocking the subsequent steps in ciliogenesis. However, unlike the mouse cells this is not associated with the absence of the centriolar appendage proteins ODF2 and CEP164.

## Discussion

Here we describe a family with two affected fetuses with compound heterozygous mutations in *C2CD3*. This is the third family to be reported with ciliopathy features caused by *C2CD3* mutations, and the causality of these mutations is further supported by *C2cd3* mouse and chick mutants that display phenotypic hallmarks of ciliary dysfunction^21^,^25^.

The previous two families were reported in a single study and presented with the classical oral, facial and digital features of OFDS^20^. However, additional features (microcephaly and cerebral malformations including cerebellar defects such as molar tooth sign) suggested a new form of OFDS (OFD14, MIM615948). Of note, skeletal features (apart from polydactyly) were not reported in these previous cases^20^. In contrast, the subjects in the current study had a different presentation, with a predominantly skeletal phenotype in addition to facial dysmorphism and pre-axial polydactyly. Neither fetus had microcephaly, considered characteristic for OFD14, although both displayed some cerebellar abnormalities, and post-mortem examination of G3P1 revealed additional CNS involvement including polymicrogyria and hydrocephalus. The differential diagnosis included SRPS and JATD, although overlap with OFD4 or Mohr-Majewski syndrome was noted. These findings illustrate that mutations in *C2CD3* can cause a much broader phenotype than OFD14, including severe skeletal dysplasia. It is possible that these differences in phenotype result from differing effects of the reported variants on C2CD3 function, due to either their location or to the nature of the mutations. One of the previously reported individuals harboured a homozygous early truncating mutation assumed to be a null allele (Fig. 2b, case 1), while the other was compound heterozygous for a frameshift and a missense mutation affecting the C-terminal C2 domains (Fig. 2b, case 2)^20^. The individuals in the present study were compound heterozygous for an early truncating mutation and a missense mutation in the N-terminal non-canonical C2 domain. Further studies will be required to determine if there is a clear genotype-phenotype correlation. Alternatively, the differing phenotypes both within and between families may result from the widely reported effect of genetic and/or epigenetic modification that occurs across the spectrum of ciliopathies^34^,^35^.

The cases presented here highlight the broad phenotypic overlap among many of the ciliopathies. The recent explosion in molecular diagnosis for ciliopathies is revealing the difficulties associated with providing a definitive clinical diagnosis, and in some cases calls into question the wisdom of continuing to divide these disorders into different sub-types as opposed to recognising that some form a continuous phenotypic spectrum. This study also broadens the molecular profile associated with predominantly skeletal phenotypes in ciliopathies. To date, skeletal ciliopathies such as SRPS and JATD have primarily been associated with mutations in genes that encode IFT proteins or the motors that drive this specialised trafficking system^36^. Amongst the exceptions to this are reported mutations in the genes encoding the centrosome-associated proteins NEK1 in SRPS^37^, CEP120 in JATD^38^ and CSPP1 in individuals with features of both JATD and another ciliopathy, Joubert syndrome^39^. Our finding of *C2CD3* mutations associated with skeletal dysplasia further links these anomalies to the earliest steps of ciliogenesis, in this case involving abnormal centriolar structure or function.

**Figure 5:**
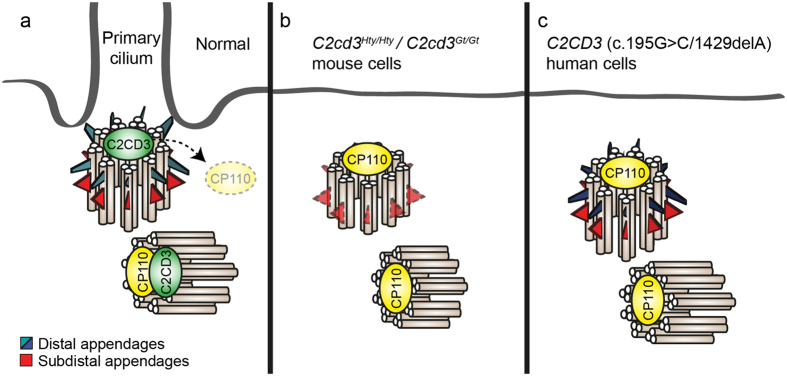
Schematic model for C2CD3-deficient mouse and human cells. (**a**) In normal conditions, the centrosome moves to the cell surface and the mother centriole with subdistal and distal appendage proteins docks to the membrane. Prior to docking CP110 is removed from the distal end of the mother centriole, a step essential for axonemal extension. (**b**) In C2CD3-deficient mouse cells, the mother centriole does not form distal appendages or anchor to the plasma membrane, and shows defective removal of CP110^20^,^23^. (**c**) In C2CD3-deficient human G3P1 cells, the subdistal and distal appendage proteins are localised normally at the mother centriole and basal body, but CP110 is not efficiently removed, hence blocking axonemal extension and ciliogenesis.

In terms of cellular phenotype, this is the first study to directly assess human cells harboring disease-causing mutations in *C2CD3*. Consistent with reported mouse and chick models^20^,^23^,^24^, these cells ultimately have a defect in their ability to extend a primary cilium. In control cells centriolar appendages form, CP110 is removed from the mother centriole, and the axoneme is extended to form a functioning cilium (schematised in Fig. 5a)^32^. In addition to its role as a regulator of centriolar length, studies in mouse cells have determined that C2CD3 is required for proper distal appendage assembly, which in turn mediates ciliary vesicle docking, CP110 removal and recruitment of a sub-set of ciliary proteins^20^,^23^. Accordingly, *C2cd3*-mutant mouse cells display defective centrosomal localisation of IFT88 and mother centriole uncapping, as well as mislocalisation of the sub-distal and distal appendage markers OFD2 and CEP164 (Fig. 5b), although in another study *C2cd3*^*Gt/Gt*^ mouse cells display normal sub-distal appendages^23^. Whether the role of C2CD3 in centriolar elongation is related to distal appendage assembly, and hence underlies the ciliary defect in these cells is yet to be determined. Like both the *C2cd3*^*Hty/Hty*^ and *C2cd3*^*Gt/Gt*^ mouse cells, the human *C2CD3*-mutant fibroblasts in this study show inefficient removal of CP110 from the distal end of the mother centriole, and defective recruitment of the core IFT component IFT88 to the centrosome (Fig. 5c). However, in contrast to the mouse models, we find that centriolar appendage proteins CEP164 and ODF2 are localised normally to the mother centriole of most *C2CD3*-mutant human cells (Fig. 5c). This suggests that the presence of these proteins is not sufficient to allow correct localisation of IFT88 and removal of CP110 from the mother centriole in a *C2CD3*-mutant context. Given that CP110 removal is now thought to occur prior to docking of the mother centriole to the membrane and conversion to a basal body^8^, our data raise the possibility that the basal body may not be correctly docking to the membrane in most G3P1 cells, but this requires further analysis at the ultrastructural level.

At the cellular level, the phenotypic differences between the *C2cd3*-mutant mouse cells and the human OFDS cells in this study may shed more light on C2CD3 function. The fact that the distal appendage protein CEP164 is localised normally in *C2CD3*-mutant human cells suggests that the function of C2CD3 in ciliogenesis could be independent of its role in distal appendage assembly, possibly through its known role in binding IFT88^23^. Alternatively, the nature of the mutations in this family may allow recruitment of CEP164 to the mother centriole but the resultant distal appendages may not be fully functional. Indeed it has been shown that distal appendage assembly involves the highly co-ordinated recruitment of at least five known proteins (including CEP164), all of which are required for the subsequent steps in ciliogenesis^32^. It is therefore possible that the sequential assembly and/or function of the distal appendages are disrupted in the G3P1 cells, despite the normal localisation of CEP164. This would be more consistent with the known role of C2CD3 in distal appendage formation uncovered in mutant mouse cells^20^,^23^, but requires further detailed analysis to determine unequivocally.

Our data provide further evidence that mutations in *C2CD3* cause a ciliopathy in humans and expand the previously reported phenotype resulting from C2CD3 dysfunction. Our analysis of human C2CD3-deficient fibroblasts shows an overall defect in ciliogenesis in line with previous studies in cells derived from loss-of-function mouse models, but reveals differences in the recruitment of proteins to the subdistal and distal centriolar appendages that are essential for basal body maturation and subsequent axonemal extension. This provides insight into the role of C2CD3 at the earliest stages of ciliogenesis, and highlights the importance of direct functional analysis of naturally occurring human mutations in precisely defining the hierarchical steps required to build a fully functional primary cilium.

## Materials and Methods

### Family recruitment and ethics

The family was recruited when referred to the Department of Clinical Genetics, Aarhus University Hospital, Denmark. This study was approved by University of Queensland Human Research Ethics Committee (approval ID 2011000876) and all methods were conducted in accordance with approved guidelines. The family gave informed written consent for whole exome sequencing and publication of images.

### Whole exome sequencing (Wes)

DNA was obtained from both fetuses and from parental blood samples, using standard methods. Whole exome sequencing was performed on genomic DNA from one affected fetus (G3P1). Briefly, DNA libraries were prepared using the Illumina TruSeq DNA sample preparation kit v2 (San Diego, CA) and exome capture was performed using the Nimblegen SeqCap EZ Human v3.0 Exome Enrichment Kit (Basel, Switzerland). Quality control and quantification was performed using Agilent 2100 Bioanalyser DNA 1000 chip kits (Santa Clara, CA) and qPCR. Massively parallel sequencing was carried out using the Illumina HiSeq 2000/2500 platform generating 100 base pair paired-end reads.

### Whole exome sequencing analysis

De-multiplexing, base calling, sequence alignment, variant calling and annotation were performed as previously described^40^. Variants that did not meet quality control and/or were attributable to platform-related artefact were excluded from further analysis. Coding variants of potentially damaging consequence (“nonsynonymous SNV”, “splicing”, “frameshift substitution”, “stopgain SNV”, “stoploss SNV”) with minor allele frequency <0.005 (based on NCBI dbSNP (release 135), 1000 Genomes^41^, 1000 Genomes small indels (called using the DINDEL programme)^42^, the SNPs of 46Genomes release by Complete Genomics, and whole exome data from over 2000 samples run internally using similar capture technology), were retained. As this family was non-consanguineous and the disorder appeared recessively inherited (both in the literature and in this family), compound heterozygosity was considered the most likely mode of inheritance. Residual variants were empirically filtered for compound heterozygous mutations. The data were also analysed for homozygous inheritance; and for single heterozygous variants in known ciliopathy genes (to assess the possibility of causative compound heterozygous mutations in another ciliopathy gene but with failure to detect a second variant due to poor coverage). See Table 1 for variant filtering. Mutation nomenclature is based on *C2CD3* RefSeq NM_015531.

### Cell culture and cilia induction

Fibroblasts were cultured in DMEM supplemented with 10% fetal bovine serum (FBS) or EGM-2 Bulletkit medium (Lonza, CC-3162) supplemented with 12% FBS, and passaged onto glass coverslips in 24-well plates. Ciliogenesis was induced by growing cells in medium with reduced serum (0.2%) for 24 hours. Cells were rinsed with phosphate-buffered saline (PBS) once and then fixed in either 100% ice-cold methanol or 4% paraformaldehyde in PBS. Cells were washed in PBS three times, then stored at 4 °C until use. Normal human juvenile foreskin-derived dermal fibroblasts (NHDF) were used as controls (PromoCell: C-12300); in the absence of a normal fetal fibroblast control, equivalent ciliogenesis was confirmed in NHDF and a fibroblast line derived from a 14-week fetus with a non-ciliopathy disease (fetal akinesia). All analyses were performed in cells maintained at low passage numbers.

### Immunofluorescence, antibodies and imaging

Fixed cells were washed once in PBS and permeabilised in PBTX (PBS + 0.1% Triton-X100) for 10 min, washed in PBS once and then 3 times in blocking buffer (PBS + 2% BSA (Roche Diagnostics, #10735086001). Primary antibodies were diluted in blocking buffer and cells incubated for 60 min at room temperature. Cells were then washed in blocking buffer three times, and then incubated with secondary antibodies diluted in blocking buffer for 60 min at room temperature. Nuclei were stained with 4′,6-diamidino-2-phenylindole (DAPI, Sigma-Aldrich, D9542) [1 mg/ml] diluted 1:4000 in PBS. Cells were washed thoroughly in PBS and mounted using DAKO anti-fading mounting medium (DAKO, S3023). Imaging was performed using an Olympus IX71 Personal Deltavision Deconvolution microscope or a Zeiss LSM-710 upright confocal microscope. Antibodies: acetylated alpha tubulin (mouse, Sigma-Aldrich, T6793) 1:5000, Arl13b (mouse, NeuroMab, 75–287) 1:300, gamma-tubulin (mouse, Sigma-Aldrich, T6557) 1:500, IFT88 (rabbit, Proteintech, 13967-1-AP) 1:300, CEP164 (rabbit, Sigma-Aldrich, SAB3500022) 1:200, ODF2 (rabbit, Sigma-Aldrich, HPA001874) 1:200, CP110 (rabbit, Proteintech, 12780-1-AP) 1:300, anti-mouse Alexa-594 (Life Technologies, A-11032) 1:500, anti-rabbit Alexa-488 (Life Technologies, A-11034) 1:500.

### Cell counting/quantification

For quantification, cells were imaged on an Olympus BX51 brightfield fluorescent microscope. Counts were performed on cells from 3 independent experiments, plated on at least two separate coverslips per experiment. Over 100 cells were counted per experiment, and graphs show the mean value across 3 independent experiments. Statistical analysis used a Student’s t-test in GraphPad Prism software. Error bars represent standard error of the mean (SEM).

## Additional Information

**How to cite this article**: Cortés, C. R. *et al.* Mutations in human *C2CD3* cause skeletal dysplasia and provide new insights into phenotypic and cellular consequences of altered C2CD3 function. *Sci. Rep.* **6**, 24083; doi: 10.1038/srep24083 (2016).

## Supplementary Material

Supplementary Information

## Figures and Tables

**Table 1:** Summary of identified variants for fetus G3P1.

	G3P1
Number of variants identified (MAF ≤ 0.05)	55294
Number of variants after QC (including exclusion of platform-related artefact and poor quality variants)	3744
Remaining variants of potentially damaging consequence^ after filtering as above	632
Novel or rare (MAF < 0.005) variants after filtering as above	347
Genes with two or more novel or rare (MAF < 0.005) variants after filtering as above	7*

(MAF = minor allele frequency, QC = quality control).

^^^Potentially damaging consequence defined as “nonsynonymous single nucleotide variants [SNV]”, “splicing”, “frameshift substitution”, “stopgain SNV”, “stoploss SNV”.

^*^FAM160B1, FCF1, OVGP1, TTN, KIAA1109, PDK4 and *C2CD3*.
