# Supplementary material for: Mutations in human C2CD3 cause skeletal dysplasia and provide new insights into phenotypic and cellular consequences of altered C2CD3 function
Source: Sci Rep. 2016 Apr 20;6:24083. doi: 10.1038/srep24083 (PMC4837335; doi:10.1038/srep24083)
Supplement: Supplementary Information [file srep24083-s1.pdf]

SUPPLEMENTARY INFORMATION

Mutations in human C2CD3 cause skeletal dysplasia and provide new insights into phenotypic and cellular consequences of altered C2CD3 function

Claudio R Cortés, Aideen M McInerney-Leo, Ida Vogel, Maria C. Rondón Galeano, Paul J Leo, Jessica E Harris, Lisa K Anderson, Patricia A Keith, Matthew A Brown, Mette Ramsing, Emma L Duncan, Andreas Zankl and Carol Wicking

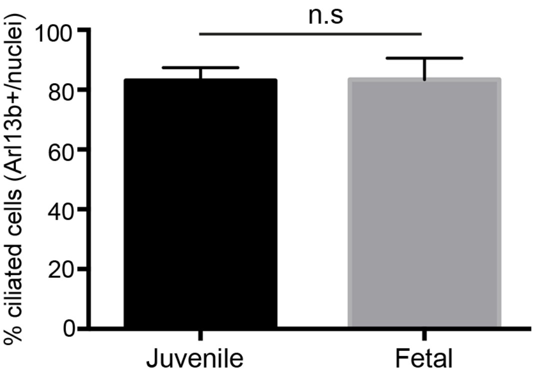

Supplementary Fig. 1. Cultured juvenile and fetal fibroblasts display the same ability to ciliate following serum starvation. The percentage of cells harboring a primary cilium was calculated by assessing Arl13b stained cilia relative to DAPI positive nuclei in cultures of normal human juvenile dermal fibroblasts (NHDF) and those derived from a 14-week fetus with a non-ciliopathy disease (fetal akinesia). This demonstrates that ciliogenesis is not overtly affected by gestational stage/age. n.s, not significant, error bars show SEM.
